# Prevention and treatment of SHIVAD8 infection in rhesus macaques by a potent d-peptide HIV entry inhibitor

**DOI:** 10.1073/pnas.2009700117

**Published:** 2020-08-20

**Authors:** Yoshiaki Nishimura, J. Nicholas Francis, Olivia K. Donau, Eric Jesteadt, Reza Sadjadpour, Amanda R. Smith, Michael S. Seaman, Brett D. Welch, Malcolm A. Martin, Michael S. Kay

**Affiliations:** ^a^Laboratory of Molecular Microbiology, National Institute of Allergy and Infectious Diseases, NIH, Bethesda, MD 20892;; ^b^Department of Biochemistry, University of Utah School of Medicine, Salt Lake City, UT 84112;; ^c^Navigen, Inc., Salt Lake City, UT 84108;; ^d^Center for Virology and Vaccine Research, Beth Israel Deaconess Medical Center, Boston, MA 02215

**Keywords:** HIV entry inhibitor, d-peptide, NHP HIV model, HIV prevention, HIV treatment

## Abstract

Cholesterol-PIE12-trimer (CPT31) is a potent d-peptide HIV entry inhibitor that targets the highly conserved gp41 N-peptide pocket region. CPT31 exhibited strong inhibitory breadth against diverse panels of primary virus isolates. In a simian-HIV chimeric virus AD8 (SHIVAD8) macaque model, CPT31 prevented infection from a single high-dose rectal challenge. In chronically infected animals, CPT31 monotherapy rapidly reduced viral load by ∼2 logs before rebound occurred due to the emergence of drug resistance. In chronically infected animals with viremia initially controlled by combination antiretroviral therapy (cART), CPT31 monotherapy prevented viral rebound after discontinuation of cART. These data establish CPT31 as a promising candidate for HIV prevention and treatment.

## Significance

This paper characterizes a d-peptide HIV entry inhibitor, CPT31, in rhesus macaques. This nonhuman primate animal model provides the most robust evaluation of an in vivo antiviral prior to human trials. As described here, CPT31 is a promising HIV drug candidate because of its high potency and breadth, long half-life, and strong activity in this macaque model (both as a preventative and therapeutic agent). The efficacy data described here complete CPT31’s preclinical studies, with clinical trials scheduled to start this year. These data also help establish d-peptides as an emerging class of therapeutics.

Combination antiretroviral therapy (cART) has greatly improved the length and quality of life of HIV-infected individuals with access to treatment and has reduced HIV transmission from treated patients (1–4). Despite continuing improvements, cART remains costly, has toxic side effects, and requires daily administration. Additionally, the high mutation rate of HIV-1 results in the rapid development of resistance to all Food and Drug Administration (FDA)-approved antiretroviral drugs (5). As a result, there is an ongoing need for novel, cost-effective therapeutic options with unique mechanisms of action and the potential for extended dosing.

We previously described the development and characterization of a d-peptide entry inhibitor, cholesterol-PIE12-trimer (CPT31), which exhibits low-picomolar (pM) potency against a tier 2 HIV-1 strain (HIV-1_JRFL_) and has a favorable pharmacokinetic (PK) profile in nonhuman primates (NHPs) (6, 7). PIE12 is a 16-residue d-peptide (composed of d-amino acids) that binds to the highly conserved gp41 trimer “pocket” region, which plays a key role in mediating viral membrane fusion (8). A trimeric version of PIE12, connected via flexible polyethylene glycol (PEG) linkers (PIE12-trimer), binds with high avidity to the three gp41 pockets in the HIV-1 envelope trimer and was previously shown to have a very high affinity for trimeric gp41 (9). In tissue-culture virus-passaging studies, resistance to PIE12-trimer was mediated by a gp41 pocket region mutation (typically Q577R) (9, 10). Conjugation of PIE12-trimer to cholesterol (to produce CPT31) localizes the inhibitor to the membrane sites of viral entry, further enhancing potency (6, 7).

We have previously used the rhesus macaque and R5-tropic simian-HIV AD8 (SHIVAD8) system as a surrogate for human HIV-1 infections because it exhibits multiple clinical features observed in the human disease, following either intravenous (i.v.) or intrarectal (i.r.) inoculation (11–13). Unlike most other SHIVs, SHIVAD8 generates sustained levels of viremia, resulting in the slow and continuous loss of CD4^+^ T lymphocytes, the development of associated opportunistic infections and lymphomas, weight loss, and death within 2 to 4 y. Virus replication in animals challenged with an infectious molecular clone derived from SHIVAD8, designated SHIVAD8-EO, can be transiently controlled with broadly neutralizing antibody monotherapy, but resistant viral variants invariably and rapidly emerge (14). In this study, we report that CPT31 is efficacious as both a preventative and a therapeutic agent against SHIVAD8-EO infections of rhesus macaques.

## Results

### CPT31 Inhibitory Breadth.

**Fig. 1.**
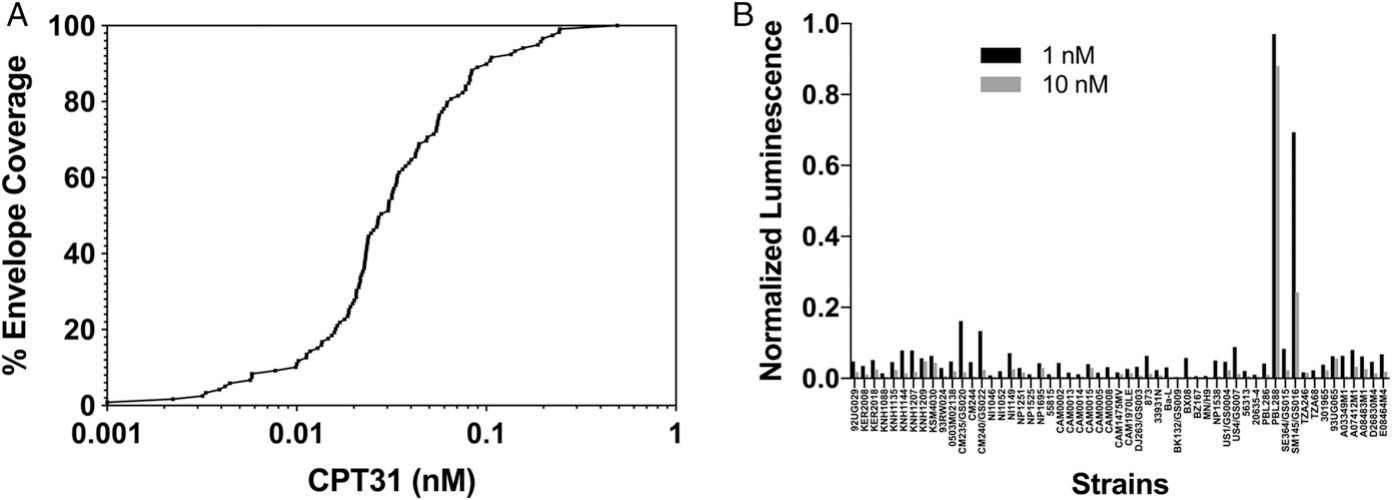
Inhibitory breadth of CPT31 against panels of HIV-1 isolates. (*A*) Percentage of strains inhibited at least 50% (IC_50_) at the indicated concentrations using the CAVD 118-strain HIV-1 pseudovirion panel. (*B*) Normalized luciferase values from a 60-strain international panel of replication-competent HIV-1 strains obtained from the NIH AIDS Reagent Program and used at 1 and 10 nM CPT31.

The inhibitory breadth of CPT31 was initially characterized using the CAVD (Collaboration for AIDS Vaccine Discovery) 118-strain pseudovirion panel (15, 16). This group of pseudovirions includes strains from clades A, B, C, D, and G, as well as circulating recombinant forms AC, ACD, AE, AG, BC, and CD. All 118 HIV-1 pseudovirions were fully inhibited by CPT31, with IC_50_s (50% inhibitory concentration) varying from <1 to 490 pM (average 50 pM) (Fig. 1*A* and *SI Appendix*, Table S1). None of the strains in this HIV-1 Env panel carried the Q577R gp41 resistance mutation.

The inhibitory breadth of CPT31 was also evaluated using an international panel of 60 primary replication-competent isolates (no. 8129) obtained from the NIH AIDS Reagent Program, and the infectivity of these viruses was assessed in TZM-bl cells. This panel includes 60 isolates from clades A, B, C, and D and circulating recombinant forms AE and AG. Three of the 60 panel viruses failed to produce luciferase levels above background and were excluded from further study. Of the 57 remaining HIV-1 strains, 55 (96%) were >90% inhibited at 10 nM CPT31, and 53 (93%) were >90% inhibited at 1 nM CPT31 (Fig. 1*B* and *SI Appendix*, Table S2). The two HIV-1 strains (PBL288 and SM145) exhibiting weak inhibition at 10 nM carry the previously described CPT31 gp41 resistance mutation (Q577R) (9). The inhibitory CPT31 breadth exhibited against HIV-1 isolates from both panels, combined with its favorable PK profile in NHPs (6), encouraged us to assess the efficacy of CPT31 in controlling SHIVAD8-EO infections of nonhuman primates.

### Prevention of Virus Acquisition by CPT31 in Rhesus Macaques.

**Fig. 2.**
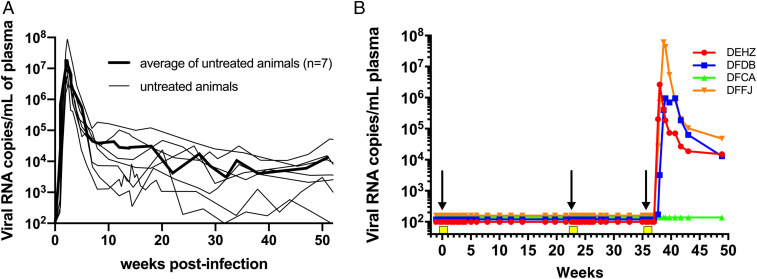
CPT31 is able to block virus acquisition in rhesus macaques. (*A*) Levels of plasma viremia in seven previously described control rhesus macaques following intrarectal inoculation with 1,000 TCID_50_ of SHIVAD8-EO (12). The average viral load in these seven animals is indicated by the bold line. (*B*) Four monkeys were inoculated intrarectally with 1,000 TCID_50_ of SHIVAD8-EO during weeks 0, 22, and 35 and intramuscularly administered 3.0, 0.5, or 0.125 mg/kg CPT31 daily, from day −3 to day +7, at each time of virus challenge. Arrows indicate the times of viral challenges; the 10 d of the three CPT31 treatments are shown as yellow boxes.

Results from multiple studies have shown that i.r. inoculation of 1,000 TCID_50_ (tissue culture infectious dose that infects 50% of cells) of the uncloned SHIVAD8 swarm virus stock or its molecularly cloned SHIVAD8-EO derivative (12, 13) results in the successful establishment of infection in rhesus macaques. Representative virus replication profiles of SHIVAD8-EO, as measured by the levels of plasma viral RNA, are shown in Fig. 2*A* (12). To assess the capacity of CPT31 to block the establishment of SHIVAD8-EO infections, four monkeys were initially treated with intramuscular (i.m.) CPT31 injections (3 mg⋅kg^−1^⋅d^−1^) for 10 d. On day 3 of therapy, the animals were challenged with 1,000 TCID_50_ SHIVAD8-EO by the i.r. route. As shown in Fig. 2*B*, all four macaques remained uninfected for 21 wk, as measured by RT-PCR analyses of sequential plasma samples. To verify that SHIVAD8-EO acquisition had not occurred in the protected monkeys, whole blood from these four animals was pooled and inoculated into a single naïve rhesus macaque. Specifically, peripheral blood mononuclear cells (PBMCs) and plasma, isolated from 20 mL of blood from each of the four protected monkeys at week 18 postchallenge, were pooled and infused i.v. into a naive animal. This macaque failed to become infected, as monitored by multiple RT-PCR assays of plasma during a 40-wk observation period.

The same four animals were then administered a second 10-d course of a lower CPT31 dose (0.5 mg⋅kg^−1^⋅d^−1^), initiated at week 22 postchallenge, and challenged again i.r. 3 d after starting this second course with 1,000 TCID_50_ of SHIVAD8-EO. The administered CPT31 again prevented virus acquisition for an additional 13 wk (Fig. 2*B*). Finally, the same four animals were treated with an even lower dose of inhibitor (0.125 mg⋅kg^−1^⋅d^−1^) for 10 d beginning at week 35 and inoculated i.r. a third time with 1,000 TCID_50_ of SHIVAD8-EO 3 d after starting this third course. In this case, plasma viremia was measured beginning at week 37 in three of the four monkeys. One animal remained uninfected at week 49 when the experiment was terminated. SHIV RNA was isolated and PCR amplified from the plasma of the three infected animals to ascertain whether resistant variants had emerged. Viral RNA from all three animals was >99% wild type (WT) at position Q577 (>100,000 reads each), indicating that insufficient CPT31 dosing, not drug resistance, was responsible for the viremia observed in these monkeys. Taken together, these data suggest a minimum protective dose for CPT31 in the high-dose rectal challenge SHIVAD8-EO/rhesus macaque model is between 0.5 and 0.125 mg⋅kg^−1^⋅d^−1^.

CPT31 plasma levels were measured in all four animals during the “dose-deescalation” experiments, using a liquid chromatography-mass spectrometry (LC-MS) bioanalytical assay (*SI Appendix*, Fig. S1). For the 3, 0.5, and 0.125 mg⋅kg^−1^⋅d^−1^ treatments, the average drug levels for samples taken immediately prior to each day’s injection were 347, 94, and 25.5 nM, respectively. The SHIVAD8-EO–protective CPT31 plasma concentration was therefore between 94 and 25.5 nM. The decay of drug levels after discontinuation of each dosing was also monitored (*SI Appendix*, Fig. S1). CPT31 levels fell below the detection limit of 1 nM in 7 wk, 11 d, and 4 d for the 3, 0.5, and 0.125 mg⋅kg^−1^⋅d^−1^ dosing regimens, respectively. These drug levels are similar to those predicted by the subcutaneous pharmacokinetic parameters previously reported in cynomolgus monkeys (6).

### CPT31 Treatment during Chronic SHIVAD8-EO Infections of Rhesus Macaques.

**Fig. 3.**
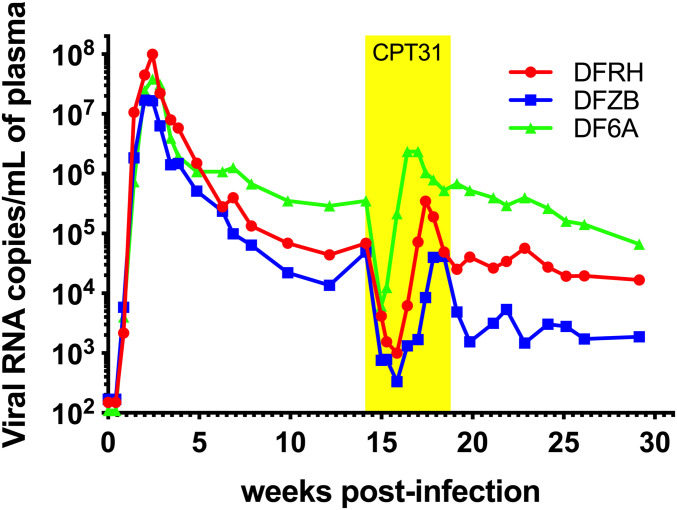
CPT31 monotherapy of chronically SHIVAD8-EO–infected rhesus macaques. Three chronically SHIVAD8-EO–infected animals were treated for 4 wk with 3 mg⋅kg^−1^⋅d^−1^ CPT31, beginning at week 14 PI.

Chronic virus infections were established in three rhesus monkeys following the i.r. inoculation of 1,000 TCID_50_ of SHIVAD8-EO. Peak levels of virus production in the 10^7^ to 10^8^ viral RNA copies per milliliter plasma range were measured at week 2 postinfection (PI) and set points of plasma viremia in the range of 10^4.1^ to 10^5.6^ viral RNA copies per milliliter were established between weeks 10 and 14 in the three animals (Fig. 3). A 30-d course of CPT31 monotherapy (3 mg⋅kg^−1^⋅d^−1^; i.m.) was initiated at week 14 PI. All three monkeys experienced a rapid ∼2-log decline in viral load within 1 to 2 wk of treatment initiation, indicating that CPT31 was able to potently suppress virus replication in vivo. However, virus rebound occurred in all three animals a week or two later. Because drug levels monitored in the three monkeys indicated that plasma CPT31 concentrations remained at the 200 nM level during the 30-d period of inhibitor administration (*SI Appendix*, Fig. S2), the emergence of CPT31-resistant virus seemed likely.

**Fig. 4.**
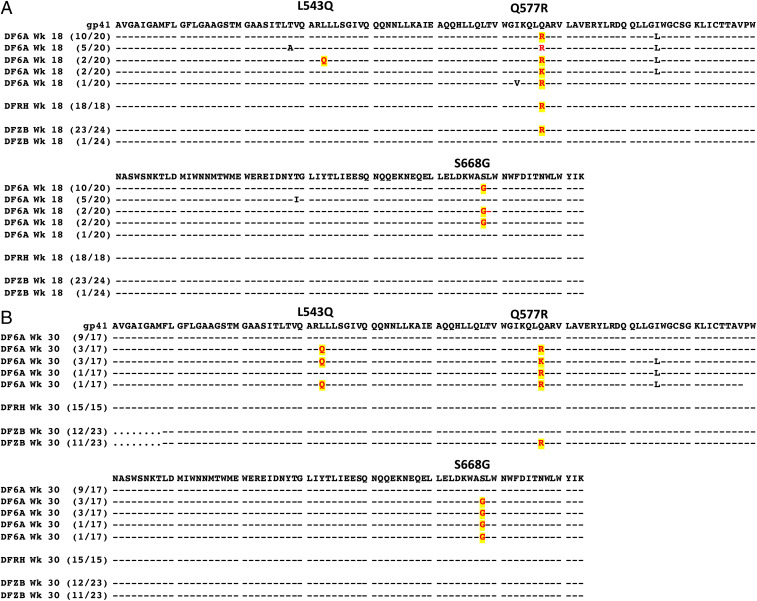
Sequence analyses of gp41 gene segments in SHIVAD8-EO chronically infected macaques treated with CPT31 monotherapy. Viral RNA was amplified by RT-PCR from plasma collected during (week 18; *A*) and following (week 30; *B*) CPT31 treatment. Common amino acid changes at positions 543, 577, and 668 of gp41 in the three animals and their frequencies are highlighted. Residues 511 to 683 are shown.

To ascertain whether resistance to CPT31 had, in fact, occurred during antiviral monotherapy, plasma was collected from the three treated macaques at weeks 18 and 30 PI, viral RNA was RT-PCR amplified, and *env* genes were sequenced. As shown in Fig. 4*A*, virtually all of the virus circulating in the three animals at week 18 PI, when SHIVAD8-EO had rebounded during CPT31 treatment, carried the Q577R CPT31 resistance substitution previously reported to arise in tissue-culture passaging studies (9, 10). Interestingly, 14 of 20 *env* genes cloned from macaque DF6A at week 18 PI had a downstream S668G change, located in the membrane-proximal external region (MPER) of gp41, in addition to the Q577R substitution.

At week 30 PI, the Q577R change had completely reverted to WT in animal DFRH (Fig. 4*B*). In contrast, 11 of 23 cloned *env* genes amplified from the plasma of macaque DFZB at week 30 PI retained the Q577R substitution. The SHIVAD8-EO circulating in monkey DF6A at week 30 PI was genetically more complex: Eight of 17 of the amplified *env* genes retained the Q577R substitution as well as the S668G change; 7 of these same 8 *env* genes had also acquired a new L543Q change (Fig. 4*B*), located in the N-heptad repeat region of gp41.

### Biological Properties of SHIVAD8-EO CPT31-Resistant Variants.

**Fig. 5.**
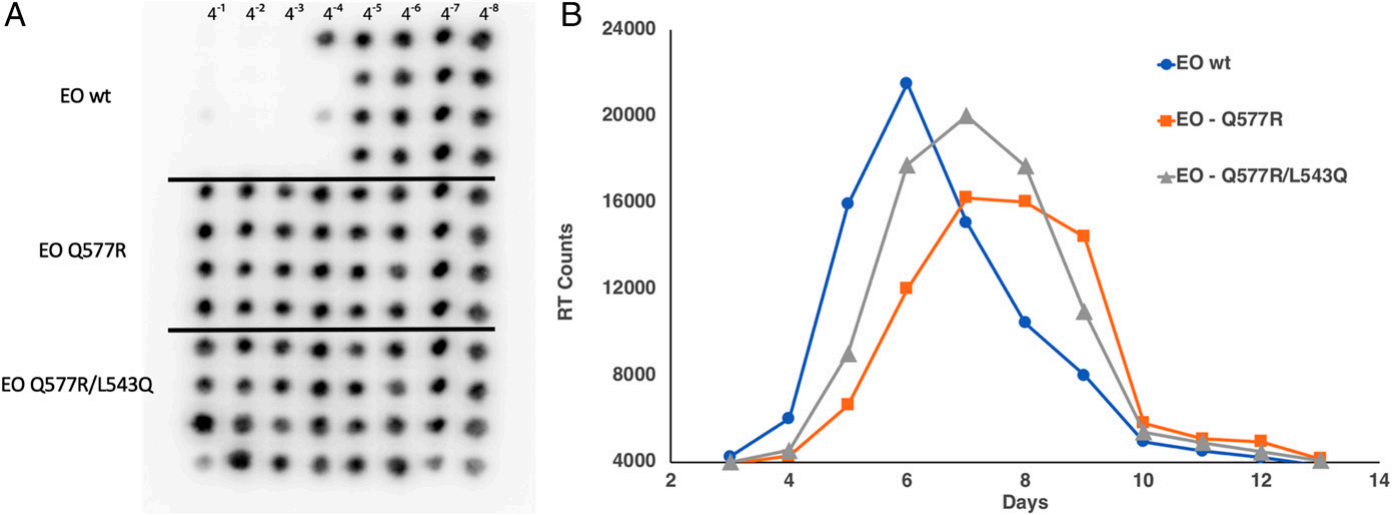
Characterization of SHIVAD8-EO CPT31-resistant variants. (*A*) End-point titrations of CPT31 inhibitory activities were assessed against wild type or resistant SHIVAD8-EO variants in rhesus PBMCs. The presence or absence of progeny virus production, treated with the serially diluted CPT31 (fourfold), was measured autoradiographically by ^32^P-RT assays performed on aliquots of the culture supernatant from day 21 of infection. The black spots in the autoradiograms indicate the presence of virion-associated reverse-transcriptase activity (i.e., no blocking of virus replication). (*B*) Replication of wild type and SHIVAD8-EO variants in rhesus monkey PBMCs. Virus stocks prepared in rhesus PBMCs were used to infect rhesus PBMCs (MOI 0.002). Virus replication was assessed by RT activity released into the culture medium.

Molecularly cloned derivatives of SHIVAD8-EO, carrying the *env* gene Q577R (AD8-577R) or Q577R plus L543Q (AD8-577R/543Q) substitutions, were constructed and used to generate virus stocks by transfecting 293T cells. The resulting supernatants were used to infect rhesus PBMCs to generate infectious virus stocks. The sensitivity of WT SHIVAD8-EO, plus the AD8-577R– and AD8-577R/543Q–resistant variants, to CPT31 was evaluated using in vitro infectivity assays with fourfold serial dilutions of the d-peptide, as described in *Methods*. The autoradiograms in Fig. 5*A* indicate that infection of WT SHIVAD8-EO was 50% blocked at the 4^−4^ dilution (1.6 nM CPT31), whereas the AD8-577R and AD8-577R/543Q SHIV variants, which emerged during the d-peptide treatment in vivo, were both resistant at the highest concentration (100 nM) tested.

The infectivities of WT SHIVAD8-EO and the AD8-577R and AD8-577R/543Q SHIV variants were next assessed using a multiplicity of infection (MOI) of 0.002 of each virus stock spinoculated onto 1 × 10^6^ rhesus PBMC cultures. As shown in Fig. 5*B*, the replication of the AD8-577R SHIV variant was delayed by 1 to 2 d compared with WT SHIVAD8-EO, and the AD8-577R/543Q SHIV variant was delayed by 1 d. Taken together, the amino acid substitutions conferring CPT31 resistance had modest effects on SHIVAD8-EO replication fitness in vitro. This result was consistent with the robust SHIV replication kinetics observed in all infected macaques following virus rebound (Fig. 3), including monkey DF6A, in which nearly half of the circulating virus population was carrying the Q577R and L543Q variants at week 30 PI.

### CPT31 Monotherapy Controls Virus Replication in Chronically SHIVAD8-EO–Infected Macaques When Administered following Virus Suppression Conferred by Prior Conventional Combination Antiretroviral Treatment.

**Fig. 6.**
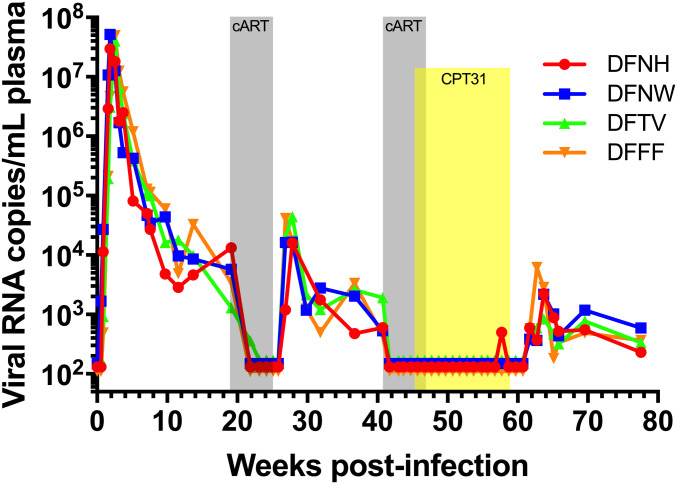
CPT31 monotherapy is able to control viremia in chronically virus-infected macaques if SHIVAD8-EO replication is first suppressed by combination antiretroviral treatment. Four monkeys were treated with cART (emtricitabine/tenofovir and raltegravir) starting at week 19 for 5 wk (gray) and at week 41 PI for an additional 6 wk. CPT31 (3 mg⋅kg^−1^⋅d^−1^) was added in the last week of cART (at week 46 PI) and continued as monotherapy for an additional 12 wk (yellow).

The transient effect of CPT31 monotherapy administered to chronically SHIVAD8-EO–infected macaques with set point levels of viremia in the 4 to 5 log_10_ range shown in Fig. 3 was not unexpected, given the high levels of sustained production of progeny virus in these animals. We wondered whether potentially potent properties of the d-peptide in vivo might be revealed in the context of an established chronic infection if virus replication was initially suppressed with conventional cART and the efficacy of CPT31 monotherapy was assessed following cessation of preceding and partially overlapping ART. This possibility was evaluated by first treating four chronically SHIVAD8-EO–infected macaques with a 5-wk course of cART (emtricitabine/tenofovir/raltegravir) starting at week 19 PI. As shown in Fig. 6, this therapy resulted in the rapid decline of plasma viremia to background levels in all four monkeys, demonstrating that a conventional cART regimen effectively suppresses SHIVAD8-EO replication. Virus was allowed to rebound for 15 wk following cessation of cART treatment at week 24 PI. At week 41 PI, a 6-wk course of cART was initiated but, in this case, CPT31 (3 mg⋅kg^−1^⋅d^−1^) was started in the last week of cART treatment (at week 46 PI) and continued for an additional 13 wk (to week 59 PI). Fig. 6 shows that virus replication was suppressed to background levels during the period of CPT31 monotherapy, and rapid rebound of plasma viremia occurred within 3 wk in all four treated animals following cessation of daily d-peptide administration. The measurement of plasma CPT31 concentrations during and after the 12 wk of inhibitor monotherapy is shown in *SI Appendix*, Fig. S3, and viral rebound occurs as expected with the drop in CPT31 to below therapeutic levels. Sequencing of the *env* genes in viruses emerging between weeks 62 and 64 following the discontinuation of d-peptide treatment revealed that 1) macaques DFFF, DFNH, and DFNW carried none of the previously observed CPT31 resistance substitutions and 2) monkey DFTV had 9/21 *env* clones with the Q577R change, 7 of which also carried the 543Q substitution (*SI Appendix*, Fig. S4).

## Discussion

In this study, we used the SHIV/macaque model to investigate the efficacy of CPT31 in both therapeutic and preventative contexts. CPT31 conferred complete protection against a high-dose rectal challenge when dosed at 0.5 mg⋅kg^−1^⋅d^−1^ and was partially protective at 0.125 mg⋅kg^−1^⋅d^−1^. Allometric scaling between macaques and humans predicts a threefold reduction in the drug dosing required to obtain comparable serum levels (17). Therefore, we hypothesize that <<0.125 mg⋅kg^−1^⋅d^−1^ dosing would protect against more physiologic (lower-dose) mucosal transmission in humans. Since CPT31 is not a component of existing cART regimens, development of CPT31 resistance as a consequence of previous participation in PrEP (e.g., in patients with undocumented infections) would be unlikely to affect cART treatment options.

The broad efficacy of CPT31 against diverse panels of HIV-1 isolates shown in Fig. 1 suggests it will be effective against a wide range of viral strains. The protective breadth of CPT31 indicates potent inhibition of all strains tested across diverse clades except for those bearing the gp41 Q577R resistance mutation. We anticipate that CPT31 will be ineffective against group O strains (9), which have a high prevalence of the Q577R primary resistance mutation (>99% Q577R in group O strains) vs. <2% Q577R in group M with similar low abundance across all major clades (Los Alamos National Laboratory HIV Sequence Database; hiv.lanl.gov/, 2018 web alignment of 6,966 representative sequences). The impact of this limitation is expected to be minimal given the relatively low prevalence of group O infections worldwide (18).

CPT31 monotherapy of an established infection rapidly inhibits viral replication but, as expected for a highly active compound in the context of uncontrolled viremia, induces drug resistance. The primary resistance mutation observed in macaques (Q577R) is in agreement with that reported in prior in vitro studies (9). The additional gp41 mutations observed in vivo (in the MPER [S668G] and N-terminal heptad repeat [L543Q]) are of unknown significance and were not observed in the context of in vitro resistance to PIE12-trimer (10). These secondary mutations have also not been reported in the context of resistance to other HIV entry inhibitors. Their effect on CPT31 resistance is likely to be indirect (e.g., affecting fusion kinetics, gp41 pocket accessibility, and/or compensating for fitness defects associated with the Q577R substitution). Additionally, most of the strains in our 118-strain CAVD pseudovirus panel contain Q543 (with only 11 having L543), and there is no significant difference in CPT31 IC_50_ between these groups. We also monitored whether CPT31 monotherapy alone could maintain the previous ART-mediated suppression of virus replication in chronically infected animals. Such CPT31 monotherapy potently controlled viral replication for an additional 12 wk, at which point CPT31 was discontinued.

Overall, the preclinical data presented support CPT31 as a strong candidate for both PrEP and as a component of combination therapy against a wide variety of HIV isolates. Our current potency and PK results suggest that CPT31 would be suitable for depot formulation and, ideally, could be used as a long-acting injectable for monthly or less frequent dosing. Studies are under way to formulate CPT31 in a depot that could be used in such a context. Additionally, this formulation could be paired with other long-acting drugs, such as nanocrystals of cabotegravir (GSK744; GlaxoSmithKline) and/or rilpivirine (TMC278; Tibotec) (19, 20), ibalizumab (21), or promising antibodies and small-molecule inhibitors (Merck’s islatravir/MK-8591 and Gilead’s GS-6207) in clinical development (22, 23). To fully evaluate CPT31’s therapeutic potential, it will be important to evaluate its efficacy when administered with other antiretrovirals to identify optimal combinations. The FDA recently cleared CPT31’s investigational new drug application, and a first-in-human trial is planned for 2020.

## Methods

### Animal Experiments.

Eleven male and female rhesus macaques (*Macaca mulatta*) of Indian genetic origin ranging from 2 to 4 y of age were maintained in accordance with the *Guide for the Care and Use of Laboratory Animals* (24) and housed in a biosafety level 2 facility; biosafety level 3 practices were followed. Phlebotomies, intrarectal virus inoculations, intramuscular drug administration, and sample collections were performed as previously described (13, 25, 26). All animals were negative for the major histocompatibility complex class I Mamu-A*01, Mamu-B*08, and Mamu-B*17 alleles.

The origin and preparation of the tissue culture-derived SHIVAD8-EO stock have been previously described (12). Animals were challenged with 1,000 TCID_50_ of SHIVAD8-EO intrarectally, as previously described (27).

CPT31 was formulated at 10 mg/mL in phosphate-buffered saline (50 mM sodium phosphate, 150 mM NaCl, pH 7.4) and administered i.m. on a mg/kg basis. Four animals were treated with a three-drug ART regimen comprising two nucleoside reverse-transcriptase (RT) inhibitors (tenofovir [PMPA] and emtricitabine [FTC]) and one integrase inhibitor (raltegravir [RAL]), starting at week 19 for 5 wk and starting at week 46 PI for 6 wk. PMPA and FTC were administered intramuscularly once a day at dosages of 20 and 40 mg/kg, respectively. RAL was administered orally (mixed with food) at a dosage of 200 mg twice a day.

### Quantitation of Plasma Viral RNA Levels.

Viral RNA levels in plasma were determined by qRT-PCR (ABI Prism 7900HT Sequence Detection System; Applied Biosystems) as previously described (25).

### Lymphocyte Immunophenotyping.

Ethylenediaminetetraacetate (EDTA)-treated blood samples were stained for flow cytometric analysis for lymphocyte immunophenotyping as previously described (11).

### Virus Sequencing.

Viral RNA was isolated from macaque plasma using the QIAmp Viral RNA Isolation Kit (QIAGEN) according to the manufacturer’s protocol and converted to complementary DNA (cDNA) using the SuperScript III or SuperScript IV First-Strand cDNA Synthesis Kit (Thermo Fisher). cDNA (2 µL) was subjected to PCR amplification for the d-peptide–binding region using Platinum PCR Supermix HiFi (Thermo Fisher) 5′-TGT​ATG​CCC​CTC​CCA​TCA​GA-3′ (forward) and 5′-CAA​GCG​GTG​GTA​GCT​GAA​GA-3′ (reverse) primers (Integrated DNA Technologies) in a 50-µL reaction. Initial denaturation was carried out at 94 °C for 2 min, followed by 32 cycles of 20 s at 94 °C, 30 s at 55 °C, and 2 min at 68 °C, with a final extension for 7 min at 68 °C. PCR products were ligated into PCR4-TOPO-TA vectors using the TOPO-TA Cloning Kit (Thermo Fisher) according to the manufacturer’s instructions. Two microliters of each ligation reaction was transformed into TOP10 chemically competent *Escherichia coli* (Thermo Fisher) according to the manufacturer’s instructions. Transformants were plated on Luria broth (LB) agar plates containing 100 µg/mL ampicillin and allowed to incubate overnight at 37 °C. Single colonies were inoculated into 3-mL cultures containing LB medium with 100 µg/mL ampicillin and allowed to incubate overnight at 37 °C with 250 rpm shaking. Each bacterial culture (2 to 3 mL) was pelleted and plasmid DNA was extracted and purified using the QIAPrep Spin Miniprep Kit (QIAGEN) according to the manufacturer’s instructions. Two micrograms of each clone was sent to the Laboratory of Molecular Microbiology Core of the National Institute of Allergy and Infectious Diseases for Sanger sequencing. Sequences were aligned using MacVector sequence analysis software.

Next-generation sequencing was conducted by using viral RNA isolated from three monkey plasma samples (DEHZ 5/21/2016, DFFJ 6/1/2016, and DFDB 6/3/2016). The isolated RNA samples, using the E.Z.N.A. Viral RNA Kit (Omega), were reverse transcribed using SuperScript IV (Thermo Fisher) and then amplified using nested PCR with primers specific to SHIVAD8 Env as well as individual barcodes for deep sequencing. The amplified samples were analyzed at the DNA sequencing core at the University of Utah using the Ion Torrent PGM Next-Generation Sequencing Platform.

### Construction of CPT31-Resistant Viruses.

The Q577R gp41 change was introduced into pSHIVAD8-EO via site-directed mutagenesis using 5′p-TCAAGCAGCTCCGGGCAAGAGTCC-3′ (forward) and 5′p-TGCCCCAGACTGTGAGTTGCAACA-3′ (reverse) with Platinum SuperFi PCR Master Mix (Thermo Fisher) as described in the manufacturer’s protocol. The PCR products were circularized using the Phusion T4 Ligase and Rapid Ligation Buffer according to the Phusion Site-Directed Mutagenesis Kit Protocol. A molecular clone containing both the Q577R and L543Q gp41 mutations was constructed by site-directed mutagenesis of pCMV-CK15 (12) using the Phusion Site-Directed Mutagenesis Kit (Thermo Fisher) as described in the manufacturer’s protocol (for the Q577R substitution) and Platinum SuperFi Master Mix (Thermo Fisher) (for the L543Q substitution). The Q577R mutation was introduced using the previously described primers, and the L543Q mutation was introduced using 5′p-GGCCAGACAATTATTGTCTGGTAT-3′ (forward) and 5′p-TGTACCGTCAGCGTTATTGACGCT-3′ (reverse) primers. PCR products were circularized using the Phusion T4 Ligase and Rapid Ligation Buffer according to the Phusion Site-Directed Mutagenesis Kit protocol. Two microliters of the ligation reaction was transformed into TOP10 chemically competent *E. coli* (Thermo Fisher), colonies were cultured, and plasmid DNA was extracted as described in the previous section. The mutant virus stocks were prepared as previously described (12).

### Virus Replication Assay in Rhesus Monkey PBMCs.

The preparation and infection of rhesus monkey PBMCs have been described previously (28). Briefly, macaque PBMCs, stimulated with concanavalin A and cultured in the presence of recombinant human interleukin-2, were spinoculated (1,200 × *g* for 1 h) with virus at the desired TCID_50_. Virus replication was assessed by RT assay of the culture supernatant as described above.

### In Vitro Blocking Assays with CPT31 in Rhesus PBMCs.

The in vitro potency of CPT31 was assessed using a 21-d PBMC replication assay (29). Briefly, CPT31 was serially diluted (fourfold, starting at 400 nM), and an aliquot of each CPT31 dilution was added to activated rhesus PBMCs (1 × 10^5^ cells per well) in quadruplicate. PBMCs were incubated for 15 min and then infected with 100 TCID_50_ of wild-type SHIVAD8-EO or CPT31-resistant SHIVAD8-EO variants. Infected cultures were maintained for 2 wk, and virus replication was monitored by ^32^P-RT assays (30). Any infectious SHIV generated during the 2 wk of incubation in PBMCs would be amplified to levels detectable by this assay.

### CPT31 Breadth In Vitro.

The inhibitory potency of CPT31 was tested against a diverse international panel of 60 primary replication-competent HIV-1 isolates obtained from the NIH AIDS Reagent Program (no. 11412) (31). Viruses were used as provided without further propagation. Infectious titers were determined using TZM-bl reporter cells (obtained from the NIH AIDS Reagent Program, no. 8129). Cells were grown to ∼90% confluency in a 96-well plate prior to the addition of serially diluted virus to achieve luminescence signals of 30,000 to 1,000,000 (BMG Labtech PolarStar Optima plate reader at maximum gain). For low-titer isolates, undiluted virus was used (up to a maximum of 10% of the medium volume). Briefly, media with 10 nM, 1 nM, or no CPT31 (uninhibited control) were added to the cells. Virus was subsequently added (up to 10% viral supernatant by volume) to achieve uninhibited luminescence signals of >30,000 (BMG Labtech PolarStar Optima plate reader at maximum gain) and incubated at 37 °C for 30 h. The medium was then removed, cells were lysed using Glo Lysis Buffer (Promega), and luciferase substrate (Bright-Glo; Promega) was added as previously described (32). Normalized luminescence values were determined by subtracting background luminescence values (TZM-bl cells with no virus) and dividing by the background-subtracted uninhibited control (1.0, uninhibited; 0, fully inhibited). Three viral strains from this panel were excluded due to insufficient signal (<30,000) in this assay (57128, TZBD9/11, and E13613M4).

The 118-strain CAVD pseudovirion panel (15, 16) was used to measure inhibitor breadth using TZM-BL indicator cells as described.

### CPT31 Plasma Concentration Measurements.

CPT31 plasma levels were measured using an LC-MS bioanalytical assay similar to the protocol previously described (6). All measurements were made using an Agilent Infinity 1290 high-performance liquid chromatography (HPLC) system and Agilent 6545A quadrupole time-of-flight mass spectrometer with a dual Jet Spray source. Samples (200 µL) were taken from flash-frozen plasma samples (stored at −80 °C). Plasma proteins were precipitated by the addition of 500 µL 2% NH_4_OH in acetonitrile. After centrifugation to remove precipitated plasma proteins, 500 to 650 µL of supernatant was transferred to a Waters Oasis MAX 96-well plate (mixed-mode strong anion exchange) and washed with 500 µL 2% NH_4_OH in water followed by 500 µL methanol. CPT31 was eluted using 2 × 25 µL 2% formic acid in methanol (for PrEP samples) or 2 × 25 µL or 50 µL 6% formic acid in methanol for the ART rebound samples and treatment samples, respectively.

One to three microliters of this sample was loaded onto an Agilent Accucore 150 C4 HPLC column (2.1 × 50 mm, 2.6-µm pore size). CPT31 was eluted using a gradient between buffer A (20 mM ammonium bicarbonate, pH 7.9, in water) and buffer B (acetonitrile). The column was run at 40 °C at 0.45 mL/min. Plasma samples were spiked with an internal standard, CPT31-IS (heavy version of CPT31 with Gly appended to the N terminus of each of the three PIE12 d-peptides in CPT31; +171.1 Da compared with CPT31; 5 to 25 nM). CPT31 and CPT31-IS were monitored using their +6 ions (*m/z* 1508.5791 and 1537.0899, respectively). The lower limit of quantitation using this method was ∼1 nM. Samples were analyzed against an eight-point standard curve of CPT31 fit to a quadratic equation. All data were normalized to the IS, except for samples from the ART rebound study, in which significant IS suppression was observed at high CPT31 concentrations, and the IS was not required to generate a high-quality standard curve.

## Supplementary Material

Supplementary File

## Data Availability

All study data are included in the article and *SI Appendix*.
