# Supplementary material for: Prevention and treatment of SHIVAD8 infection in rhesus macaques by a potent d-peptide HIV entry inhibitor
Source: Proc Natl Acad Sci U S A. 2020 Aug 20;117(36):22436–42. doi: 10.1073/pnas.2009700117 (PMC7486783; doi:10.1073/pnas.2009700117)
Supplement: Supplementary File [file pnas.2009700117.sapp.pdf]

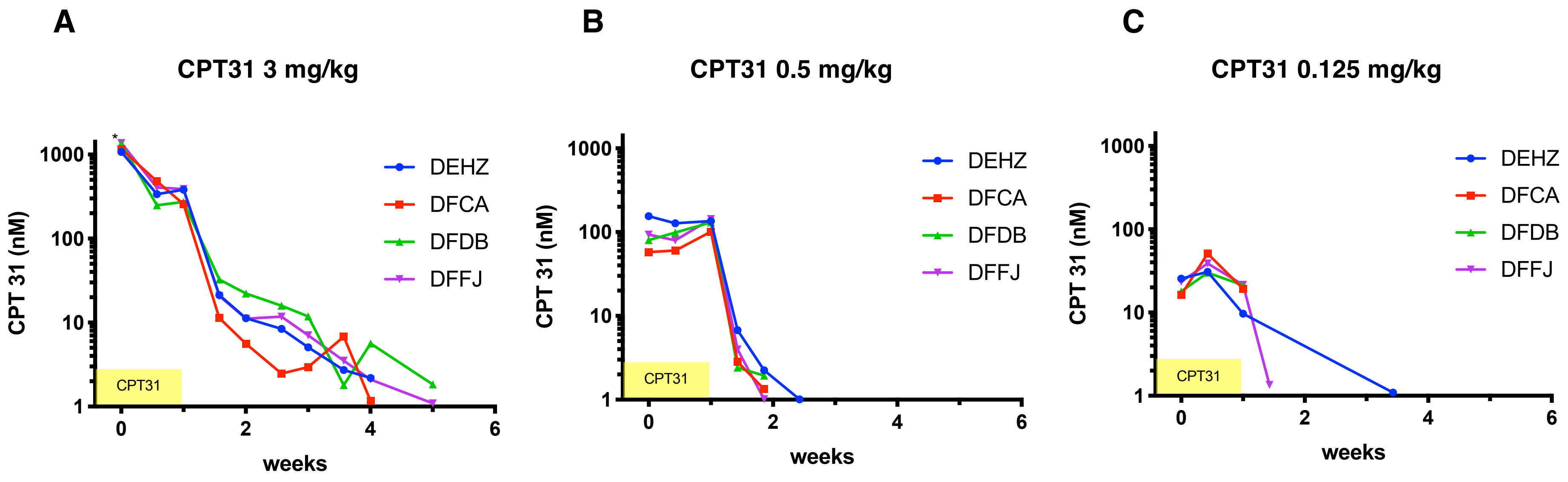

Figure S1: Pharmacokinetic measurements of CPT31 levels during virus acquisition blocking studies. Four monkeys were intramuscularly administered 3.0 (A), 0.5 (B), or 0.125 (C) mg/kg CPT31 daily, beginning on days –3 to day +7, at each time of SHIVAD8-EO challenge, respectively. Drug levels were measured using an LC-MS bioanalytical assay with an internal standard. The dosing periods are indicated in yellow.

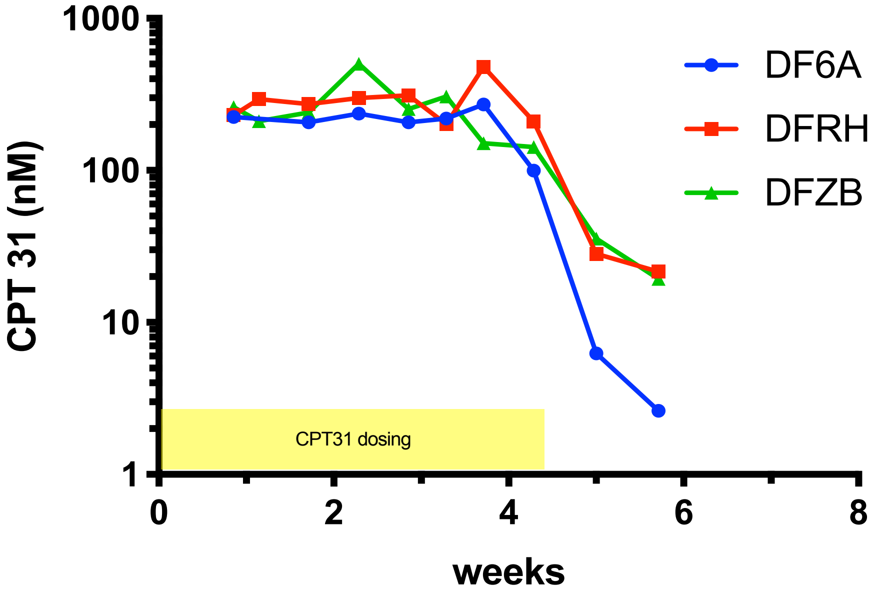

Figure S2: Pharmacokinetic measurements of CPT31 during monotherapy of chronically infected SHIVAD8-EO rhesus macaques. CPT31 was administered at 3 mg/kg/day. Drug levels were measured using an LC-MS bioanalytical assay with an internal standard. The dosing period is indicated in yellow.

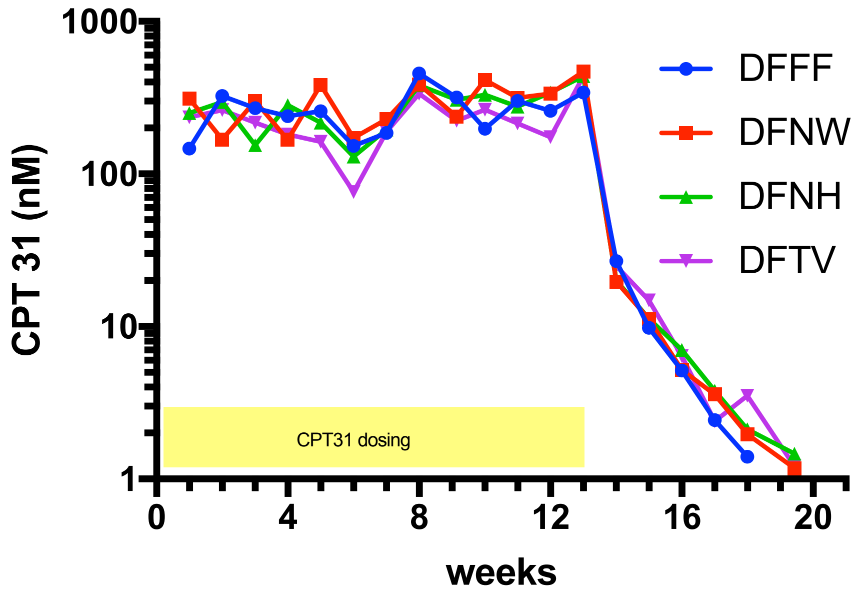

Figure S3: Pharmacokinetic measurements of CPT31 in conjunction with cART treatment/cessation in chronically SHIVAD8-EO infected rhesus macaques. CPT31 was administered at 3 mg/kg/day for 12 weeks between weeks 46 and 58 PI. Drug levels were measured using an LC-MS bioanalytical assay with an internal standard. Dosing period indicated in yellow.

```
                                                                                 Q577R
             AVGAIGAMFL GFLGAAGSTM GAASITLTVQ ARLLLSGIVQ QQNNLLKAIE AQQHLLQLTV WGIKQLQARV LAVERYLRDQ QLLGIWGCSG KLICTTAVPW
DFFF (12/21) ---------- ---------- ---------- ---------- ---------- ---------- ---------- ---------- ---------- ----------
DFFF  (3/21) ---------- ---------- ---------- ---------- ------R--- ---------- ---------- ---------- ---------- ----------
DFFF  (2/21) ---------- ---------- ---------- ---------- ---------- ---------- ---------- ---------- ---------- ----------
DFFF  (2/21) ---------- ---------- ---------- ---------- ---------- ---------- ---------- ---------- ---------- ----------
DFFF  (1/21) ---------- ---------- ---------- ---------- ---------- ---------- ---------- ---------- ---------- ----------
DFFF  (1/21) ---------- ---V------ ---------- ---------- ---------- ---------- ---------- ---------- ---------- ----------

DFNH (11/11) -------V-- ---------- ---------- ---------- ---------- ---------- ---------- ---------- ---------- ----------

DFNW (19/30) ---------- ---------- ---------- ---------- ---------- ---------- ---------- ---------- ---------- ----------
DFNW  (5/30) -----R---- ---------- ---------- ---------- ---------- ---------- ---------- ---------- ---------- ----------
DFNW  (3/30) ---------- ---------- ---------- ---------- ---------- ---------- ---------- ---------- ---------- ----------
DFNW  (3/30) ---------- ---------- ---------- ---------- ---------- ---------- ---------- ---------- ---------- ----------

DFTV (12/21) ---------- ---------- ---------- ---------- ---------- ---------- ---------- ---------- ---------- ----------
DFTV  (7/21) ---------- ---------- ---------- --Q------- ---------- ---------- ------R--- ---------- ---------- ----------
DFTV  (2/21) ---------- ---------- ---------- ---------- ---------- ---------- ------R--- ---------- ---------- ----------


             NASWSNKTLD MIWNNMTWME WEREIDNYTG LIYTLIEESQ NQQEKNEQEL LELDKWASLW NWFDITNWLW YIK
DFFF (12/21) ---------- ---------- ---------- ---------- ---------- ---------- ---------- ---
DFFF  (3/21) ---------- ---------- ---------- ---------- ---------- ---------- ---------- ---
DFFF  (2/21) ---------- ---------- -----N---- ---------- ---------- ---------- ---------- ---
DFFF  (2/21) ---------- ---------- --G------- ---------- ---------- ---------- ---------- ---
DFFF  (1/21) ---------- ---------- ---------- -----L---- ---------- -------G-- ---------- ---
DFFF  (1/21) ---------- ---------- ---------- ---------- ---------- ---------- ---------- ---

DFNH (11/11) ---------- ---------- ---------- ---------- ---------- ---------- ---------- ---

DFNW (19/30) ---------- ---------- ---------- ---------- ---------- ---------- ---------- ---
DFNW  (5/30) ---------- ---------- ---------- ---------- ---------- -------G-- ---------- ---
DFNW  (3/30) ---------- ---------- ------D--D ---------- ---------- ---------- ---------- ---
DFNW  (3/30) ---------- ---------- ---------D ---------- ---------- ---------- ---------- ---

DFTV (12/21) ---------- ---------- ---------- ---------- ---------- ---------- ---------- ---
DFTV  (7/21) ---------- ---------- ---------- ---------- ---------- ---------- ---------- ---
DFTV  (2/21) ---------- ---------- ---------- ---------- ---------- ---------- ---------- ---
```

Figure S4: Sequence analyses of gp41 gene segments in SHIVAD8-EO chronically infected macaques treated with cART and then with CPT31 monotherapy. Viral RNA was amplified by RT-PCR from plasma collected at week 62. The Q577R changes identified in found in animal DFTV are highlighted.

Table S1: Inhibitory breadth of CPT31 against the CAVD 118-strain pseudovirion panel.

Strain	Clade	CPT31 IC_{50} (pM)
191084_B7_19	A	1
211_9	A	2
928_28	A	3
0815_V3_C3	A	4
3365_V2_C20	A	4
MS208_A1	A	10
6540_V4_C1	A	10
Q23_17	A	20
Q461_E2	A	20
191955_A11	A	20
T251_18	A	20
9004SS_A3_4	A	20
Q769_D22	A	20
235_47	A	30
T278_50	A	30
263_8	A	30
3817_V2_C59	A	30
6041_V3_C23	A	40
6545_V4_C1	A	40
Q842_D12	A	50
T250_4	A	60
3415_V1_C1	A	60
T255_34	A	60
6480_V4_C25	A	80
Q259_17	A	80
0260_V5_C36	A	200
T257_31	A	240
R2184_C4	AE	3
C3347_C11	AE	20
CNE8	AE	20
C2101_C1	AE	20
R3265_C6	AE	20
R1166_C1	AE	20
CNE5	AE	30
BJOX028000_10_3	AE	30

C1080_C3	AE	40
620345_C1	AE	50
BJOX025000_01_1	AE	50
C4118_9	AE	80
BJOX010000_06_2	AE	90
BJOX009000_02_4	AE	160
BJOX015000_11_5	AE	190
AC10_29	B	10
SC422661_8	B	10
REJO4541_67	B	20
SC05_8C11_2344	B	20
62357_14_D3_4589	B	20
1012_11_TC21_3257	B	20
1054_07_TC4_1499	B	30
WEAU_D15_410	B	30
WITO4160_33	B	30
TRJO4551_58	B	30
TRO_11	B	40
6535_3	B	40
THRO4156	B	50
CAAN5342	B	60
1056_10_TA11_1826	B	60
RHPA4259_7	B	70
6240_08_TA5_4622	B	80
6244_13_B5_4567	B	100
QH0692	B	110
1006_11_C3_1601	B	140
PVO_4	B	220
ZM233_6	C	4
DU422	C	10
249M_B10	C	10
DU156_12	C	10
DU172_17	C	10
0013095_2_11	C	10
CE0393_C3	C	10
ZM249_1	C	10
ZM135_10A	C	10
CE704809221_1B3	C	20
CNE58	C	20

CNE53	C	20
ZM53_12	C	20
CAP210_E8	C	20
ZM214_15	C	20
6811_V7_C18	C	20
CNE20	C	20
ZM109_4	C	20
CE1086_B2	C	20
CNE17	C	20
CNE19	C	20
ZM197_7	C	20
CE1172_H1	C	20
CNE52	C	30
16055_2_3	C	30
CNE21	C	30
CAP45_G3	C	30
CE2060_G9	C	30
ZM247V1(REV_)	C	30
CE0682_E4	C	30
3301_V1_C24	C	40
246F_C1G	C	40
001428_2_42	C	40
1394C9G1(REV_)	C	60
CE703010054_2A2	C	60
3103_V3_C10	C	70
BF1266_431A	C	80
CNE30	C	80
16845_2_22	C	80
CE1176_A3	C	110
7030102001E5(REV_)	C	190
CE2010_F5	C	490
231966_C2	D	10
6952_V1_C20	D	20
A07412M1_VRC12	D	50
89_F1_2_25	D	60
3016_V5_C45	D	80
6405_V4_C34	D	140
231965_C1	D	240
X1254_C3	G	10

X1193_C1	G	20
X1632_S2_B10	G	20
X2088_9	G	20
P1981_C5_3	G	30
X2131_C1_B5	G	30
P0402_C2_11	G	40

Table S2: Inhibitory breadth of CPT31 against 60-strain international panel of replication-competent virus obtained from the NIH AIDS Reagent Program. Note that three strains were excluded due to insufficient titer (see Methods).

Strain	Clade	Normalized luminescence	
		1 nM CPT31	10 nM CPT31
92UG029	A	0.048	0.018
KER2008	A	0.035	0.011
KER2018	A	0.052	0.025
KNH1088	A	0.015	0.004
KNH1135	A	0.046	0.024
KNH1144	A	0.079	0.015
KNH1207	A	0.079	0.018
KNH1209	A	0.057	0.048
KSM4030	A	0.064	0.044
93RW024	A	0.03	0.007
M02138	AE	0.048	0.02
CM235/GS020	AE	0.162	0.017
CM244	AE	0.046	0.005
CM240/GS022	AE	0.134	0.024
NI1046	AE	0.009	0.003
NI1052	AE	0.02	0.003
NI1149	AE	0.071	0.026
NP1251	AE	0.03	0.016
NP1525	AE	0.012	0.002
NP1695	AE	0.043	0.03
55815	AG	0.012	0.005
CAM0002	AG	0.044	0.003
CAM0013	AG	0.016	0.003
CAM0014	AG	0.012	0.005
CAM0015	AG	0.04	0.03
CAM0005	AG	0.016	0.002
CAM0008	AG	0.032	0.003
CAM1475MV	AG	0.017	0.012
CAM1970LE	AG	0.027	0.015
DJ263/GS003	AG	0.033	0.008
873	B	0.064	0.013
33931N	B	0.024	0.009
Ba-L	B	0.031	0.002
BK132/GS009	B	0.003	0.002
BX08	B	0.058	0.005
BZ167	B	0.006	0.002
MN/H9	B	0.007	0
NP1538	B	0.05	0.006
US1/GS0004	B	0.047	0.023
US4/GS007	B	0.089	0.013
56313	C	0.021	0.007
20635-4	C	0.01	0.004
PBL286	C	0.042	0.01
PBL288	C	0.97	0.88

SE364/GS015	C	0.084	0.023
SM145/GS016	C	0.694	0.243
TZA246	C	0.017	0.016
TZA68	C	0.023	0.003
301965	C	0.039	0.023
93UG065	D	0.063	0.056
A03349M1	D	0.064	0.005
A07412M1	D	0.08	0.033
A08483M1	D	0.062	0.026
D26830M4	D	0.047	0.015
E08464M4	D	0.068	0.019
J32228M4	D	0.039	0.02
NKU3006	D	0.028	0.002
